# Advanced In Vivo Prediction by Introducing Biphasic Dissolution Data into PBPK Models

**DOI:** 10.3390/pharmaceutics15071978

**Published:** 2023-07-19

**Authors:** Alexander Denninger, Tim Becker, Ulrich Westedt, Karl G. Wagner

**Affiliations:** 1 Department of Pharmaceutical Technology, University of Bonn, Gerhard-Domagk-Strasse 3, 53121 Bonn, Germany; alexander.denninger@cordenpharma.com (A.D.); tim.becker@uni-bonn.de (T.B.); 2 Corden Pharma GmbH, Otto-Hahn-Strasse, 68723 Plankstadt, Germany; 3 AbbVie Deutschland GmbH & Co. KG, Knollstrasse, 67061 Ludwigshafen, Germany; ulrich.westedt@abbvie.com

**Keywords:** PBPK modeling, biphasic dissolution, drug product, prediction, poorly soluble drugs, enabling formulations

## Abstract

Coupling biorelevant in vitro dissolution with in silico physiological-based pharmacokinetic (PBPK) tools represents a promising method to describe and predict the in vivo performance of drug candidates in formulation development including non-passive transport, prodrug activation, and first-pass metabolism. The objective of the present study was to assess the predictability of human pharmacokinetics by using biphasic dissolution results obtained with the previously established BiPHa+ assay and PBPK tools. For six commercial drug products, formulated by different enabling technologies, the respective organic partitioning profiles were processed with two PBPK in silico modeling tools, namely PK-Sim and GastroPlus^®^, similar to extended-release dissolution profiles. Thus, a mechanistic dissolution/precipitation model of the assessed drug products was not required. The developed elimination/distribution models were used to simulate the pharmacokinetics of the evaluated drug products and compared with available human data. In essence, an in vitro to in vivo extrapolation (IVIVE) was successfully developed. Organic partitioning profiles obtained from the BiPHa+ dissolution analysis enabled highly accurate predictions of the pharmacokinetic behavior of the investigated drug products. In addition, PBPK models of (pro-)drugs with pronounced first-pass metabolism enabled adjustment of the solely passive diffusion predicting organic partitioning profiles, and increased prediction accuracy further.

## 1. Introduction

Dissolution assays represent the state-of-the-art method for guiding formulation development [1]. However, monophasic compendial dissolution methods are not sufficient for all drugs [2]. Especially in the case of poorly soluble drugs many advanced methods have been developed to increase their predictability with regard to their in vivo performance [3]. There are two possibilities to achieve a higher discriminatory power, either by modifying standard methods [3] or by applying more complex models [4,5,6].

Biphasic absorption models are promising to increase the discriminatory power of enabling formulations [3,6,7]. The biphasic dissolution model (BiPHa+) consists of a combination of 1-decanol and an optimized buffer system simulating the gastrointestinal passage as a one-vessel biphasic dissolution method. Under controlled hydrodynamic conditions (160 rpm), the relative absorption area, the ratio of organic to aqueous phase, and the selected drug concentrations are the discriminating performance factors [8]. After the liberation of the poorly soluble drug from a formulation under non-sink conditions in aqueous medium the dissolved fractions of the drug distribute into an organic absorption compartment. Sink conditions are obligatory in the absorption compartment to characterize the rate-limiting step of absorption, namely dissolution and re-dissolution of the undissolved drug in the aqueous phase [9]. Additionally, the partitioning rate into the absorption compartment has to be higher compared to (re-) dissolution [7,10]. The BiPHa+ developed in our group fulfills these requirements and demonstrated a high degree of in vivo relevance in several experiments validated by Level A in vitro–in vivo relationship (IVIVR) [8].

Physiological-based pharmacokinetic modeling (PBPK) represents an in silico tool, which mechanistically describes the behavior of a drug in vivo [11,12]. Moreover, metabolism, enterohepatic circulation, physiochemical parameters, transporters, in vitro data, or influence of ingestion can be simulated [13]. However, it is challenging to implement the results of advanced dissolution models for poorly soluble drugs into PBPK models This is a consequence of mechanistic input models that focus primarily on dissolution and precipitation. However, re-dissolution of already precipitated API could contribute significantly to the absorbable dose [8]. A major challenge of monophasic dissolution models to characterize these formulations is that they do not cover the entire dynamic in vivo processes of the drug substance. Monophasic dissolution models only simulate drug release, dissolution, and precipitation. Important processes such as re-dissolution and absorption are better simulated by the biphasic dissolution model [10,14,15]. Thus, the input of biphasic dissolution data may allow the data to be implemented more realistically in PBPK modeling.

The general subject of the present investigation was to get a rapid estimation and prediction of the in vivo performance of enabling formulations investigated by the BiPHa+ assay.

The present study describes an approach to introduce biphasic dissolution results for six different marketed drug products into GastroPlus^®^ and PK-Sim. The dissolution results were generated by applying the BiPHa+ assay as described in a previous study [8]. However, as the BiPHa+ assay was only able to characterize the passively absorbed part, a combination of a biphasic partitioning profile with GastroPlus^®^ and PK-Sim is expected to enhance the predictive power, in particular for model drugs like fenofibrate, which is a prodrug, activated by ester hydrolysis, and nimodipine, which undergoes a high degree of first pass metabolism.

## 2. Materials and Methods

### 2.1. Materials

The investigated drug products, which comprise nano- and microcrystal-based as well as amorphous solid dispersion (ASD) formulations, are shown in Table 1.

PBPK modelling was performed using GastroPlus^®^ 9.6 (Simulations Plus, Inc., Lancaster, CA, USA) and PK-Sim 7.3, which is part of the Open Systems Pharmacology Suite (Bayer Technology, Bayer AG, Leverkusen, Germany).

### 2.2. Physiochemical Characterisation

Physiochemical properties were taken from our previous work [8]. The properties of fenofibrate/fenofibric acid were determined for this study. The Sirius-T3 (Pion Inc (UK) Ltd., Forest Row, UK) was used to measure pKa values through UV-metric titration [16]. LogP values were determined by a HPLC method using a water methanol gradient [17]. Solubility was UV-metrically quantified by shaking flask method. P_eff_ was calculated using PK-Sim 7.0 (see also Section 2.4). All mentioned values are given in Table 2.

### 2.3. In Vitro Data

**Figure 1:**
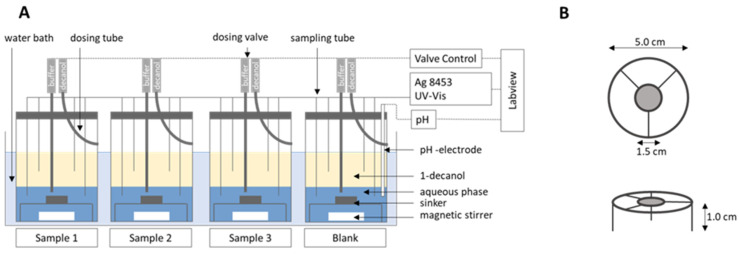
(**A**) BiPHa+ model setup, (**B**) sinker dimension. Reproduced with permission from Denninger et al., A Rational Design of a Biphasic Dissolution Setup—Modelling of Biorelevant Kinetics for a Ritonavir Hot-Melt Extruded Amorphous Solid Dispersion; Pharmaceutics, 2020 [15].

**Figure 2:**
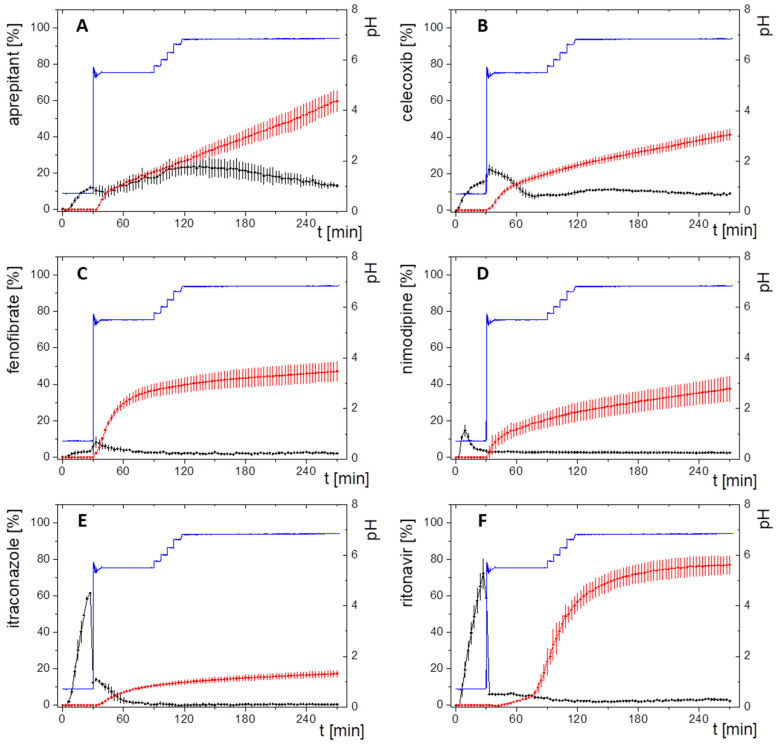
Biphasic dissolution profiles; black: drug concentration in aqueous phase; red: drug concentration in organic phase; blue: pH; (**A**) aprepitant; (**B**) celecoxib, (**C**) fenofibrate (new data); (**D**) nimodipine; (**E**) itraconazole; (**F**) ritonavir. Adapted with permission from Denninger et al., Shared IVIVR for Five Commercial Enabling Formulations using the BiPHa+ Biphasic Dissolution Assay; Pharmaceutics., 2021 [8].

The biphasic dissolution data for aprepitant, celecoxib, nimodipine, itraconazole, and ritonavir were taken from Denninger et al. [8], the data for fenofibrate were measured accordingly. All dissolution experiments were carried out fully automatically by the BiPHa+ assay mimicking fasted intestinal conditions (Figure 1). The model simulates the gastrointestinal passage in the fasted state. In the first 30 min, the formulations were allowed to dissolve in 0.1 N HCl representing the in vivo gastric emptying. To simulate the intestinal transition from gastric to intestine conditions, the pH was increased to 5.5 and then dynamically adjusted from pH 5.5 up to pH 6.8. Simultaneously, a stock solution of sodium taurocholate and lecithin was added. The absorption of drug by the organic acceptor phase (1-decanol) started at 30 min, immediately after the gastric stage. The overall duration of the experimental sequence was 4.5 h. The resulting biphasic dissolution profiles are given in Figure 2. A more detailed model description was published previously [8,15].

### 2.4. Model Development and Evaluation

PBPK models of the six different formulations of poorly soluble drugs were developed in PK-Sim 7.3 and GastroPlus^®^ 9.6 using mean pharmacokinetic data of human subjects taken from clinical trials by taking three consecutive steps:

First, the PBPK modelling software was fed with physiochemical properties of the drugs. To enter physiological properties of an average human, we used the sometimes-limited description of the trial participant’s data. Then, the available data were used to extrapolate with the database data of the respective PBPK modelling software to get the final description of the average human applied in the software. Second, elimination/distribution models including absorption, if oral human data were available, were developed for the respective model drugs. The development was performed based on clinical ADME PK data obtained after application of oral solutions or intra venous injections, which is described for each drug substance in the following Section 2.4.1, Section 2.4.2, Section 2.4.3, Section 2.4.4, Section 2.4.5 and Section 2.4.6. Finally, a pharmacokinetic prediction of each enabling formulation was calculated based on the biphasic partitioning profiles and the developed elimination/distribution models, and subsequently compared to reported plasma concentration time data. A detailed description of the model development, on which the later prediction is based, is given in the following below:

The physicochemical properties of the six model substances (Table 2) were implemented in the PBPK tools. The intestinal permeabilities of the drugs were calculated using PK-Sim based on logP instead of logMW values as recommended previously [18], where MW_eff_ (>300 g/mol) is the molecular weight and MA is the membrane affinity.

(1) $$P_{eff}=265.8\;{\cdot \;MW}_{eff}^{-4.500}\cdot MA.$$

**Figure 3:**
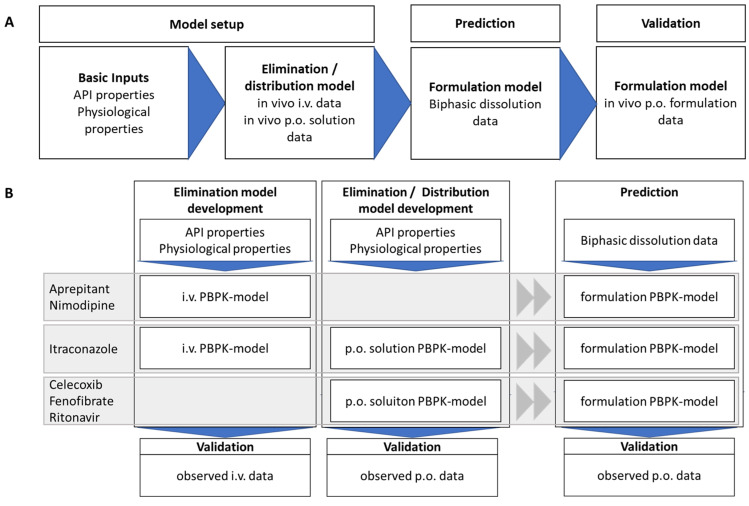
Workflow of PBPK-model development (**A**) and prediction of different drugs and their validation with observed plasma concentration time profiles from Phase 1 ADME studies. (**B**) Applied models for the different APIs depending on available literature data (details can be found in Section 2.4.1, Section 2.4.2, Section 2.4.3, Section 2.4.4, Section 2.4.5 and Section 2.4.6).

The workflow of PBPK model development for each model drug in the respective drug product is shown in Figure 3. Physiological characteristics such as age, weight, fasting state, and water intake were initially included. In essence, the kinetic parameters for drug distribution, elimination, and metabolism were derived from the available in vivo pharmacokinetic data (ADME: after intra venous (i.v.) or oral (p.o.) solution treatment) for the assessed drugs to develop accurate PBPK models. If a drug undergoes CYP metabolism, the enzyme was also considered in the identification procedure. Ideally, elimination models were developed by using plasma profiles of intravenously administered solutions. Subsequently, absorption models were implemented by using pharmacokinetic profiles of orally administered solutions. However, for drugs with low solubility intravenous plasma-concentration data were often not available. Therefore, most of the PBPK models were implemented with pharmacokinetic data from oral solutions (Figure 3). The distribution/elimination PBPK model for nimodipine and aprepitant were developed by using intravenous data. The distribution/elimination and absorption PBPK model for itraconazole was stepwise established starting from intravenous data to oral solution formulation. For celecoxib, fenofibrate and ritonavir only oral solution data were available, and therefore, distribution/elimination and absorption models were developed simultaneously. Verification of the PBPK modelling was performed using literature data from average plasma concentration-time profiles.

Basically, two methods are potentially applicable to implementing biphasic dissolution data into PBPK software.

**Figure 4:**
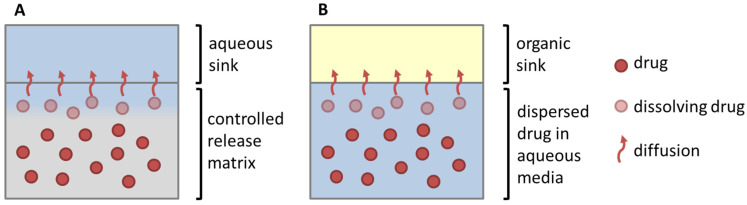
Similarity of drug dissolution from a controlled release matrix (**A**) and the partitioning of the dissolved drug from enabling formulations in the biphasic dissolution assay (**B**). The rate limiting steps represents either drug release from a modified release matrix (**A**) or dissolution of a poorly soluble drug in an aqueous media (**B**) (Adapted with permission from Wagner, Perorale Retardarzneiformen; Pharmakon 2016 [26]).

(1) The organic partitioning profile was deemed as a controlled release dissolution profile (Figure 4). The dissolution of an undissolved poorly soluble drug was assumed to be equivalent to the drug release from a controlled release formulation, which represents the rate-limiting step of absorption. The partitioning of a poorly soluble drug into the organic layer was assumed to be equivalent to the drug release of a soluble and permeable drug (Figure 4). The partitioning rate of the drug is then comparable to the in vivo fraction absorbed [8].

(2) The DDDPlus add-on tool of GastroPlus^®^ allows developing a mechanistic precipitation—dissolution model, which can be then integrated in the PBPK model (GastroPlus^®^ only). The mechanistic model parameters can be derived from biphasic dissolution data. However, the dissolution behaviours of enabling formulations are more complex and not entirely covered by the DDDPlus tool. Depending on the formulation approach, the dissolution and re-dissolution mechanism differ, as the following examples are demonstrating the complexity: Drug and polymer of an ASD-based formulation (ideally) dissolve simultaneously [19,20,21,22], whereas the dissolution (rate) of the drug of micro- or nanocrystal-based products is enabled by drug particle size reduction [23]. However, with both formulation approaches drug precipitation can occur depending on the drug and formulation properties. Thus, the dissolution rate of the primarily formulated drug might strongly differ from the dissolution rate of the precipitated drug, as the precipitate sometimes could change particle size or solid state and therefore change its dissolution kinetics [15,23]. The reasons for this could be different: During the dissolution process of an ASD, the supersaturated drug can precipitate in its crystalline or amorphous form, whereas the amorphous precipitates might be present as submicron colloids [24]. Both solid states, crystalline and amorphous could be coexistent at the same time [25]. Even the non-crystalline precipitated drug can result in different species with different dissolution properties. These differences could be caused by a transformation of the precipitated drug during the dissolution of the precipitate, e.g., reducing the particle size and increasing the dissolution rate [15]. All these processes are summarized indirectly by the organic partitioning profile using the biphasic assay.

With these considerations, the PBPK predictions were carried out by implementing the biphasic dissolution data as controlled release concentration time profiles into the previously developed models from the oral solution or intravenous formulations according to method 1.

The following sections provide further pharmacokinetic information on the assessed drugs from literature, which were considered in the development of the PBPK models, respectively.

#### 2.4.1. Aprepitant (Nanocrystal)

The nano-formulated aprepitant partitioned into the organic layer according to its formulation type following zero-order kinetics (Figure 2A). The highly lipophilic drug exhibits a high plasma protein binding of >98% [27]. The absolute oral bioavailability was reported to be approximately 60% [28]. In this study, 20 volunteers received 2 mg aprepitant intravenously. The final oral commercial formulation was administered with 227 mL of water.

#### 2.4.2. Celecoxib (Microcrystal)

Celecoxib represents a weak acid with a physiologically irrelevant pKa value of 10.7. The biphasic dissolution profile is given in Figure 2B. The estimated plasma protein binding is higher than 97% [29]. Celecoxib is mainly metabolized by CYP2C9 [29]. Absolute oral bioavailability has not been investigated in humans. However, the absolute oral bioavailability was investigated in poor metabolizing beagle dogs under fasted conditions [30]. Since metabolism was supposed to be minimal, an estimate of the fraction absorbed in the microcrystalline celecoxib formulation could be made. The fraction absorbed in poor metabolizing dogs was within the range of 40% [31]. Pal et al. investigated an oral solution in humans under fasted conditions [31]. Pharmacokinetic data of the commercial drug product Celebrex^®^ were taken from the literature [30]. Celebrex^®^ was administered in the fasted state together with 210 mL of water.

#### 2.4.3. Fenofibrate (Microcrystal)

Fenofibrate is a highly permeable prodrug, which is activated by ester hydrolysis [32]. Thus, the biphasic dissolution model characterizes only the fraction absorbed of micronized fenofibrate (Figure 2C). Its active form is fenofibric acid [32], which is used as a PK measure. In contrast, fenofibrate is not detectable in the plasma [33]. Fenofibric acid is highly bound to plasma proteins (>99%) [32]. The absolute oral bioavailability of 46% was indirectly calculated through a nano-formulation as described in the literature by comparing a nano-formulation with a micronized formulation [34,35,36]. Fei et al. compared the oral plasma profile of micronized fenofibrate and an oral solution [37].

#### 2.4.4. Itraconazole (ASD)

Itraconazole exhibits a plasma protein binding of 99.8%. It is a substrate for CYP3A4 metabolism [38] and enters the enterohepatic circulation [39]. Itraconazole was intravenously administered as a β-cyclodextrin solution, which formed the basis of the distribution/elimination model [40]. An oral solution was investigated to determine the absolute oral bioavailability in a fasted state, which allows for extending of the PBPK model with oral absorption [41]. The absolute oral bioavailability of the capsule formulation (15.6%) was estimated by comparing the relative bioavailability of the solution and the capsule formulation with a known absolute oral bioavailability as described in the literature [42]. Both, the oral solution and the HPMC-based formulation were investigated under fasted conditions in two studies by Barone et al. [38,38]. The capsule formulation was administered together with 200 mL of water.

The biphasic dissolution result of the highly lipophilic drug formulated as HPMC-based amorphous solid dispersion is given in Figure 2D.

#### 2.4.5. Nimodipine (ASD)

Nimodipine is highly permeable as demonstrated by an early C_max_ value at 1 h after oral administration. However, the predicted permeability values of 8.08 × 10^−5^ cm/min were very low [43]. Therefore, the permeability from CaCo2 assay (6.06 × 10^−5^ cm/min) was used to calculate the in vivo permeability via GastroPlus^®^ [44]. Blardi et al. investigated an i.v. formulation and the commercial drug product in a clinical study [45]. An oral solution has not been evaluated in a clinical study so far. Nimodipine is extensively metabolized by CYP3A4 liver enzymes, which explains a low oral bioavailability of 3.5%. Approximately 90% of the absorbed drug is metabolically inactivated during the first liver passage [45]. Thus, the fraction absorbed of nimodipine is in a range of 35%, considering 90% metabolism, which is in accordance with biphasic dissolution data (Figure 2E).

#### 2.4.6. Ritonavir (ASD)

The commercial drug product of ritonavir is an amorphous solid dispersion based on polyvinylpyrrolidone-vinyl acetate copolymer. In the BiPHa+ assay, ritonavir displays a sigmoidal partitioning profile (Figure 2F). CYP3A4 is the most important metabolizing enzyme of ritonavir [46]. Fraction absorbed is in the range of 60–80% [34]. Salem et al. conducted a clinical study in children by administering an oral solution formulation under moderate fat conditions, which formed the basis for the absorption/distribution and elimination PBPK model [47]. The solubility of ritonavir in FeSSIF-V2 is 18.5 µg/mL [14]. The oral pharmacokinetic profile of the commercial product was investigated by administering 100 mg ritonavir after a 10 h fasting period [48].

## 3. Results

### 3.1. Model Development

**Figure 5:**
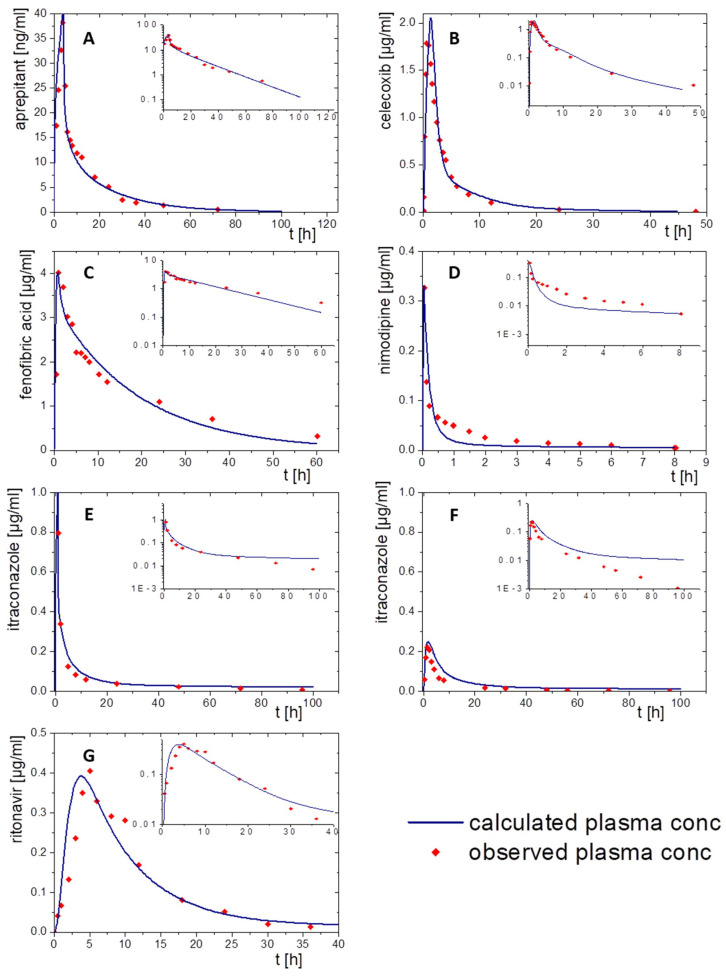
Simulated plasma concentration–time profiles using PK-Sim (blue line) and observed pharmacokinetic data (red dots): (**A**) aprepitant after a 2 h infusion, (**B**) celecoxib following an oral solution, (**C**) fenofibrate administered as oral solution, (**D**) nimodipine administered by a single injection, (**E**) itraconazole after infusion of a cyclodextrin-based formulation, (**F**) itraconazole after oral administration of a solution, and (**G**) ritonavir administered as oral solution.

**Figure 6:**
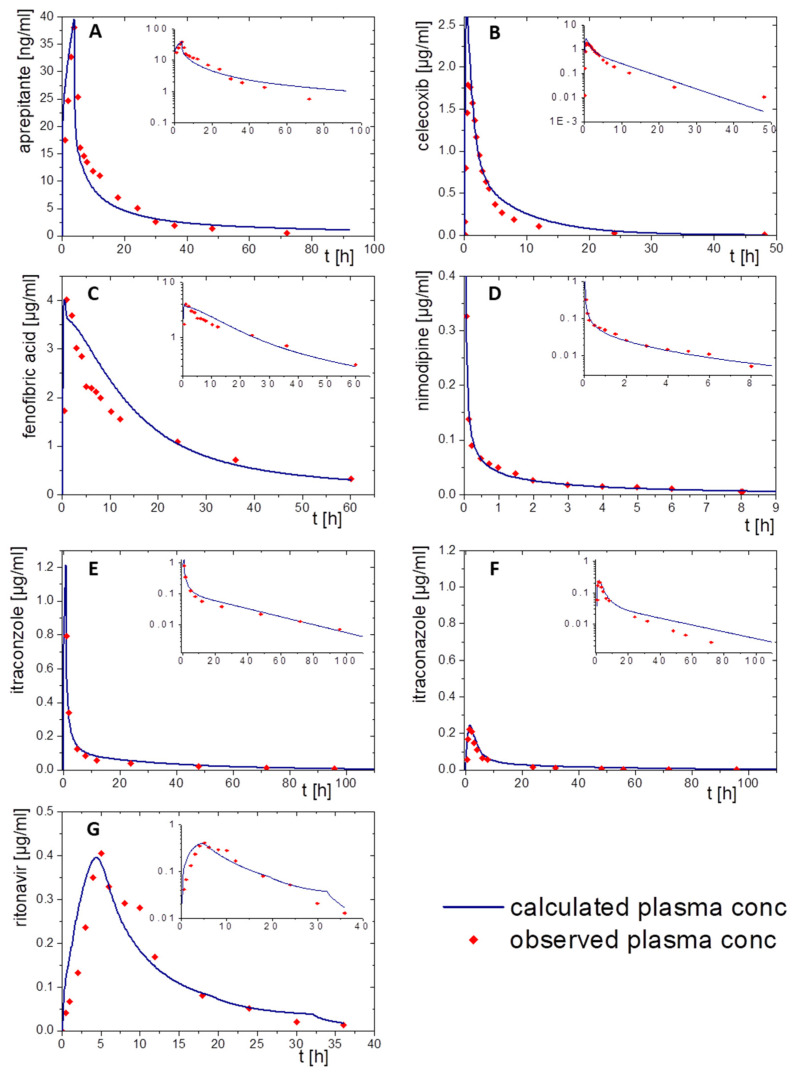
Simulated plasma concentration time profile using GastroPlus^®^ (blue line) and observed pharmacokinetic data (red dots): (**A**) aprepitant after a 2 h infusion, (**B**) celecoxib following an oral solution, (**C**) fenofibrate administered as oral solution, (**D**) nimodipine administered by a single injection, (**E**) itraconazole after infusion of a cyclodextrin-based formulation, (**F**) itraconazole after oral administration of a solution formulation, and (**G**) ritonavir administered as oral solution.

The elimination and distribution PBPK models and the subsequent oral absorption PBPK models were built depending on available data. Subsequently, the BiPha+ results (distribution kinetics into the decanol phase) were implemented in the predictive model surrogating the in vivo absorption data. In the first step, the elimination and distribution models were developed to receive an accurate PBPK model (Figure 5 and Figure 6). Ideally, elimination data derived from PK profiles following i.v. administration. However, i.v. PK-data were only available for aprepitant, nimodipine and itraconazole formulations. PK data for ritonavir, fenofibrate and celecoxib from intravenous administration were not available. Consequently, oral solutions were used for the implementation of the elimination distribution models in these cases. If an oral solution was used, the predicted permeability values of Table 2 were used for the absorption model. The models were developed and subsequently validated using average plasma concentration-time pharmacokinetic data of all individuals in the clinical trial.

The following paragraphs provide more details on the PK model input to PK-Sim and GastroPlus^®^ for each drug and describe the consistency of the modeled PK with the available in vivo data of the orally applied drug product.

Aprepitant was administered as a 2 h infusion. The distribution/elimination models described the observed data, even C_max_ values, very accurately in both modelling software packages. Especially the model in PK-Sim met the observed values very precisely (Figure 5A). In the GastroPlus^®^ model, the plasma concentration decreased rapidly during the elimination period. After that, the elimination speed increased until the modeled values were above the in vivo data from about 30 h on (Figure 6A).

Nimodipine was intravenously applied as a bolus. Only 10% of nimodipine can be detected in the venous blood, because of its high CYP3A4 metabolism (Figure 5D and Figure 6D). Thus, CPY3A4 metabolism was implemented for the intravenous application in the PBPK model to enhance model accuracy. The pharmacokinetic prediction with GastroPlus^®^ provided a higher accuracy with respect to elimination (Figure 6D), whereas the PK-Sim model resulted in a faster metabolizing rate compared to the observed data (Figure 5D).

For the pharmacokinetic assessment of celecoxib, the model was based on data from an oral solution, as data from an intravenous formulation were not available. Both PBPK models matched the observed data with high precision (Figure 5B and Figure 6B). PK-Sim predicted a change in the slope of elimination/distribution at 5 h and 15 h. In contrast, GastroPlus^®^ calculated a change of the elimination/distribution slope solely at 5 h. C_max_ values were slightly overestimated in both PBPK simulations.

The prodrug fenofibrate is activated by ester hydrolysis. Therefore, the plasma concentration–time profile was determined as its activated metabolite fenofibric acid. The model for fenofibric acid and fenofibrate was simultaneously developed to ensure an accurate description of the pharmacokinetics of the oral solution [32]. Activation, distribution, and elimination were in agreement with the observed data of the oral solution formulation using both software tools (Figure 5C and Figure 6C).

To build a model for ritonavir, the literature provided only data obtained after administration of oral solution to children at moderate fed state conditions (Figure 5G and Figure 6G). Prior to implementing the dissolution data from the BiPha+ assessment, the model was adapted to adults at fasted state conditions to predict the in vivo performance of the commercial ASD-based drug product. Small deviations from the observed plasma profile occurred during the absorption period and from 30 h on within the PK-Sim prediction. (Figure 5G). Similar deviations were observed in the GastroPlus^®^ simulation (Figure 6G). The C_max_ values (0.4 µg/mL) were precisely calculated with both models.

For itraconazole, PK data were available after administration of intravenous and oral solutions. The elimination model of the cyclodextrin-based i.v. formulation administered by infusion was established including CYP 3A4 metabolism and enterohepatic circulation. By optimizing intravenous models in PK-Sim and GastroPlus^®^, the obtained pharmacokinetic profile was consistent with in vivo data (Figure 5E and Figure 6E). The elimination rate decreased after 40 h in the PK-Sim prediction. By extending the model with the oral solution, the elimination turned out slower than the observed elimination values (Figure 5F and Figure 6F). Considering that the observed data from both studies were from different clinical trials, the models were in good agreement within the first 10 h. Nevertheless, C_max_ and t_max_ values were correctly calculated.

In sum, both in silico tools were able to describe the elimination and distribution of the assessed drugs by using in PK-Sim and GastroPlus^®^. In some cases, intravenous data were not available to develop the pure elimination/distribution model. Therefore, it was difficult to differentiate between effects resulting from absorption or elimination. However, the pharmacokinetic profiles were described with high accuracy.

### 3.2. IVIVE Using Organic Biphasic Partitioning Profiles

After developing the PBPK elimination/distribution models in GastroPlus^®^ and PK-Sim as described in the previous section, the in vitro dissolution data obtained from the six drug products were assessed towards the ability of the BiPHa+ assay to predict the in vivo performance.

**Figure 7:**
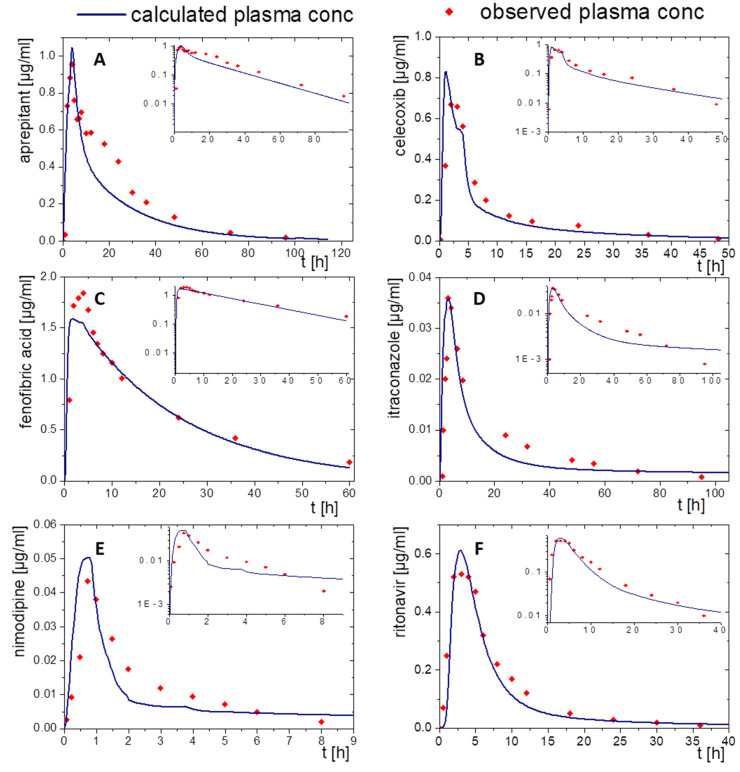
Simulated plasma concentration–time profile based on in vitro distribution data of the BiPHa+ dissolution test using PK-Sim after oral administration (blue line) and in vivo data (red dots) for the drug products of (**A**) aprepitant, (**B**) celecoxib, (**C**) fenofibrate, (**D**) itraconazole, (**E**) nimodipine, and (**F**) ritonavir.

**Figure 8:**
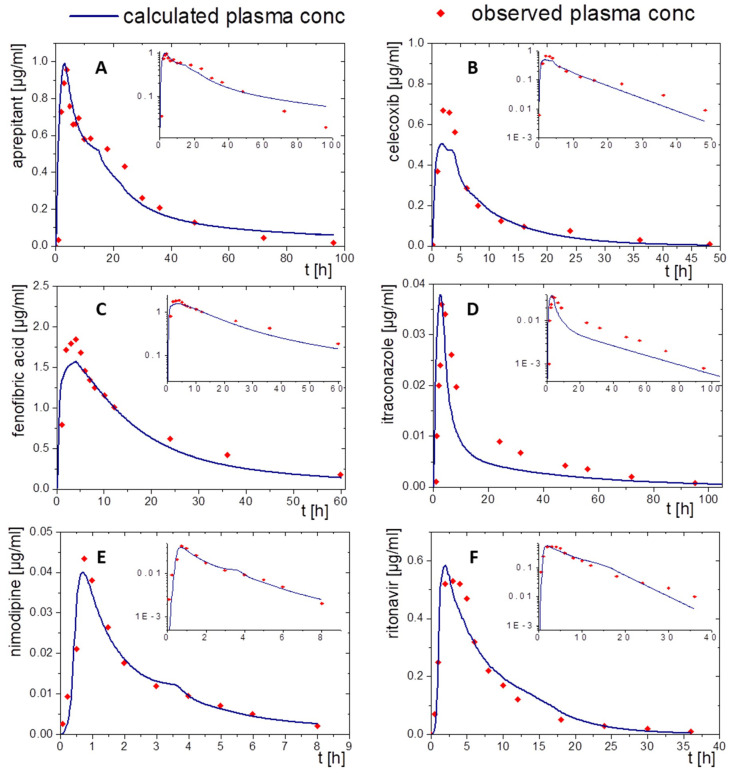
Simulated plasma concentration time profile based on in vitro distribution data of the BiPHa+ dissolution test using the GastroPlus^®^ controlled release function after oral administration (blue line) and in vivo data (red dots) for the drug products of (**A**) aprepitant, (**B**) celecoxib, (**C**) schfenofibrate, (**D**) nimodipine, (**E**) itraconazole, and (**F**) ritonavir.

For this, the drug concentration–time profiles from the organic absorption phase (decanol) together with the predicted P_eff_ values (Table 2) were fed into GastroPlus and PK-Sim as described in Section 2.4. Then, the pharmacokinetic profiles were simulated by applying the PBPK elimination/distribution models. The results are given in Figure 7 and Figure 8. Further details are provided in the following sections.

#### 3.2.1. Aprepitant (Nanocrystal)

The nanocrystal-based formulation for aprepitant displayed a zero-order partitioning profile after an initial higher partitioning rate in the organic phase up to a final concentration of approximately 60% (Figure 2A). Plasma concentration–time profiles predicted by PK-Sim and GastroPlus^®^ led to accurately predicted C_max_ and t_max_ values, which underlines the validity of the absorption model. The observed pharmacokinetic data of aprepitant indicated an enterohepatic circulation between 10 and 30 h, which was additionally implemented into the PBPK models (Figure 7A and Figure 8A). Deviations of the PK-Sim simulation from the observed pharmacokinetic data occurred (Figure 7A), because enterohepatic circulation in PK-Sim decreased the elimination rate only [49]. The enterohepatic circulation model of GastroPlus^®^ was able to predict the pharmacokinetic profile more precisely (Figure 8A).

#### 3.2.2. Celecoxib (Microcrystal)

The microcrystal-based drug product Celebrex^®^ showed zero-order in vivo absorption kinetics with an initially faster absorption rate as obtained in the biphasic dissolution assessment (Figure 2B). In particular, the simulated absorption period of celecoxib based on in vitro partitioning data occurred differently in the PK-Sim and GastroPlus^®^ simulation (Figure 7B and Figure 8B). In contrast, t_max_ and AUC were in good agreement. The course of elimination and distribution was matched by both models (Figure 7B and Figure 8B). The in vivo C_max_ value of 0.66 µg/mL was overestimated in PK-Sim (0.83 µg/mL) and underestimated in GastroPlus^®^ (0.58 µg/mL).

#### 3.2.3. Fenofibrate (Microcrystal)

A first-order and subsequent zero-order absorption process were observed in the in vitro partitioning profile (Figure 2C), reaching a maximum of approx. 45%. Thus, a very fast absorption rate was expected. The simulated t_max_ values were approximately at 5 h despite its high permeability (P_eff_, Table 2) and high in vitro partitioning rate in the BiPHa+ assay. Therefore, activation by ester-hydrolyses played an important role. Taking this activation step into account, the plasma concentration-time profiles of fenofibric acid in vivo were accurately matched by the ones obtained from PK-Sim and GastroPlus^®^ (Figure 7C and Figure 8C). C_max_ values from PK-Sim (1.59 µg/mL) and GastroPlus^®^ (1.57 µg/mL) were slightly underestimated compared to in vivo (1.89 µg/mL).

#### 3.2.4. Itraconazole (ASD)

As described in Section 2.4, the PBPK model for itraconazole was stepwise developed from an intravenous administration to an oral solution (Figure 5E,F and Figure 6E,F). The biphasic partitioning profile was implemented into the oral solution PBPK model (Figure 2C) to predict human pharmacokinetics. In vivo C_max_ value and the predicted C_max_ values from PK-Sim (36 ng/mL) and GastroPlus^®^ (38 ng/mL) were almost identical with the maximum plasma concentration in vivo (36 ng/mL). The predicted plasma concentration–time profile from PK-Sim in the first 10 h met the in vivo data. From 10 h on, where the elimination is dominant, deviations from the in vivo profiles occurred (Figure 7D). The same applies to GastroPlus^®^, which predicted a slightly lower plasma concentration over the entire elimination period starting at 10 h (Figure 8D). However, both C_max_ and t_max_ values obtained from the model predictions were in good agreement with in vivo data.

#### 3.2.5. Nimodipine (ASD)

The formulation of nimodipine represents a first generation, polyethylene glycol-based ASD, which resulted in a square root like partitioning profile (Figure 2D). Nimodipine is highly permeable but exhibits limited bioavailability because of its high first-pass effect. Therefore, the use of PBPK modelling to predict in vivo pharmacokinetics using the BiPHa+ partitioning profiles is obligatory. The combination of PBPK modelling to describe the metabolism and the organic partitioning profile as absorption profile generated meaningful pharmacokinetic predictions (Figure 7E and Figure 8E). The short elimination time and low bioavailability in the modelling were consistent with in vivo data. Overall, the two in silico tools provided sound pharmacokinetic predictions, whereby deviations were more pronounced in PK-Sim (Figure 7E).

#### 3.2.6. Ritonavir (ASD)

A sigmoidal partitioning profile was acquired from the BiPHa+ assay for the ASD-based drug product Norvir^®^ (Figure 2F). The PBPK models were successfully scaled from children in fed state to adults in fasted state. GastroPlus^®^ and PK-Sim accurately predicted the pharmacokinetic profile using the BiPHa+ partitioning profile. The predicted t_max_ and C_max_ value were highly consistent with in vivo plasma data (Figure 7F and Figure 8F).

### 3.3. Predictive Performance of PBPK Models

**Figure 9:**
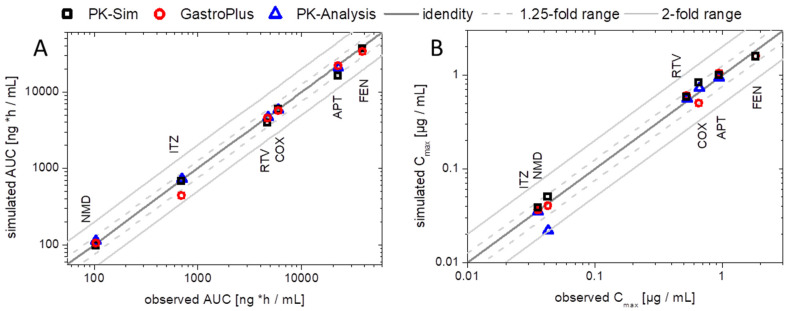
Verification of enabling formulation PBPK in GastroPlus^®^ or PK-Sim and PK models [8] including the biphasic dissolution profiles by comparing observed vs. predicted values: (**A**) AUC values and (**B**) C_max_ values. The dark grey line represents the line of identical observed in vivo and predicted. The light grey solid lines represent the 2-fold, the light grey dotted lines the 1.25-fold boundary.

The developed elimination and distribution PBPK models built the basis for the prediction of the plasma concentration–time profiles using the BiPHa+ results. The predicted profiles were then compared with in vivo data. The correlation of in vivo and predicted AUC and C_max_ values is displayed in Figure 9 to validate the prediction performance of the models using the BiPHa+ dissolution data. In sum, all PBPK model predictions were within the 50 to 200% boundaries as recommended previously [50,51].

In addition, Figure 9 also contains AUC and C_max_ values obtained from the pharmacokinetic prediction using a compartmental model without PBPK modeling (as described previously [8]). Deviations between pharmacokinetic prediction and PBPK modeling became obvious in the case of a pronounced metabolism. The deviations received from the nimodipine PK prediction were significantly decreased using the PBPK models because the high first-pass effect was considered in the mechanistic PBPK model, which led to a more precise prediction (Figure 9). PBPK modeling created the mechanistical understanding of the in vivo behavior of the drugs.

## 4. Discussion

The general subject of the investigation was to get a rapid estimate and prediction of the in vivo performance of enabling formulations based on in vitro dissolution data from the BiPHa+ assay. Pharmacokinetic predictions were successfully performed by implementing the biphasic partitioning profiles in GastroPlus^®^ or PK-Sim. The organic partitioning profiles were interpreted like controlled release dissolution profiles. Thus, dissolution was assumed as the rate-limiting step for absorption in the case of poorly soluble drugs and not as drug release from a modified release dosage form.

Because of the highly complex and partly not well understood gastrointestinal behavior of enabling formulations, many dissolution models did not entirely cover all relevant processes [11]. Several approaches have been investigated for the incorporation of dissolution data into the PBPK simulations [52]. However, the more complex a formulation is, the less a semi-mechanistic dissolution model can be utilized for a PBPK model. For example, enhanced drug dissolution can be achieved by applying the ASD approach, but the dissolution characteristics remain complex due to its potential propensity of precipitation [53]. USP methods are commonly used for the investigations of immediate-release formulations (e.g., tablets), even for BCS IV compounds [54]. Despite some successful implementations into PBPK models, this in vitro setup lacks sink conditions for compounds with very poor solubility and thus is limited to providing a comprehensive understanding of the dissolution and absorption process [55]. Moreover, important factors like pH shift, biorelevant media and the consideration of the mucus layer covering the gastrointestinal tract are not part of the USP in vitro dissolution setup [56]. For a better understanding of in vivo precipitation, biorelevant dissolution setups have been developed taking pH and biorelevant components into account and then used for PBPK modeling [57]. Nevertheless, dynamic processes like supersaturation, precipitation, and redissolution cannot be correctly displayed due to the lack of an in vitro absorption component, and thus, might be over- or underestimated in the in silico model [55]. Despite the absence of a mucus-simulating layer, which is not feasible for technical reasons, the biphasic dissolution assay allowed a more dynamic characterization of formulations by introducing an additional partitioning step [3,6,13]. Thus, the rate-limiting and thermodynamically-unfavored dissolution process could be better characterized independently from the characteristics of the undissolved drug in the aqueous phase. Consequently, many biorelevant processes during in vitro dissolution of the tested formulations were considered by implementing the partitioning profile in the PBPK software. By this, the development of a complex in silico dissolution/precipitation model was not necessary. Besides the ASD formulation principle for the drug products of itraconazole, nimodipine, and ritonavir, dissolution enhancement was achieved via particle size reduction for the marketed products of aprepitant, celecoxib, and fenofibrate. Itraconazole and ritonavir as weak bases were found to rapidly dissolve in the gastric phase (at pH 1) during biphasic dissolution. For both compounds, precipitation occurred in accordance with the pH shift to pH 5.5. Nevertheless, the partitioning sink indicated intestinal absorption of dissolved and redissolved drug. This led to an accurate prediction by incorporating the partitioning profile into the PBPK modeling. In particular, for the performance interpretation of ritonavir the biphasic dissolution deduced precipitation as nano-droplets and following Ostwald ripening of these nano-droplets as the mechanism behind the sigmoidal partitioning profile [15]. Even though, in the case of itraconazole C_max_ was predicted perfectly, the later part of the plasma concentration–time curve was clearly underestimated. Thus, elimination and distribution, deduced from i.v. and p.o. (solution) administration, were not able to entirely elucidate the plasma levels for the ASD formulation. Itraconazole was the only example, where clinical data of oral solution and an intravenous application were reported. Especially in this example, study to study variability became obvious: the identified elimination model (Figure 5F and Figure 6F) led to some deviations during the elimination period of the oral solution (Figure 5G and Figure 6G). Accordingly, it is assumed that the absorption profile was sufficiently predicted via the biphasic dissolution. Likewise, the zero-order absorption profiles of aprepitant and celecoxib were incorporated into the PBPK models. However, for these compounds, the input of dissolution alone was not able to cover the in vivo measured plasma levels. To enhance the predictive power, metabolism (celecoxib) and enterohepatic circulation (aprepitant) were implemented. Moreover, partitioning profiles of first-order (fenofibrate) and root-like (nimodipine) kinetics were determined and used for the respective PBPK models. Activation of fenofibrate to fenofibric acid by plasma esterases was successfully implemented and led to a high prediction accuracy in GastroPlus^®^ and PK-Sim (Figure 7C and Figure 8C). Although, the partitioning profile of nimodipine indicated a fraction absorbed of approx. 35%, a bioavailability of 3.5% is reported in the literature. However, by applying a CYP3A4 first-pass metabolism into the PBPK model of nimodipine, the predictions became accurate, in contrast to the simple compartmental prediction approach reported in an earlier work, where the pharmacokinetic was not sufficiently described [8]. Overall, the resulting correlation between the predicted pharmacokinetic parameters AUC and C_max_ and the observed values thereof demonstrated the predictive power of the combination of the biorelevant BiPHa+-assay and PBPK modeling considering different PBPK modeling software packages.

## 5. Conclusions

The interplay between dissolution and absorption after oral administration of enabling formulations is a critical factor for the bioavailability and, therefore, achieving respective therapeutic effects.

For a faster and more efficient investigation of potential formulation candidates, the BiPHa+ assay was established as a helpful tool to describe dynamic processes like dissolution, precipitation, re-dissolution, and absorption in a setup relevant for in vivo. The implementation of the partitioning profiles of six drug products from BiPHa+ dissolution into a PBPK model in the same way as supposedly conducted for extended-release formulations provides an accurate prediction of plasma profiles after oral administration. In this way, the development and implementation of a complex dissolution/precipitation/re-dissolution model could be avoided, without losing prediction accuracy as these effects are mimicked by the BiPHa+ model, which provides finally the organic partitioning profile. Moreover, metabolic pathways could be easily implemented extending the applicable range of predictable drugs as demonstrated for nimodipine (pronounced metabolism) and fenofibrate (activation via ester hydrolysis). A model extension to transporter-mediated absorption should be possible and a matter of future investigations.

In sum, the combination of the biorelevant biphasic dissolution and in silico PBPK modeling provides a rational approach to predict the PK behavior of poorly soluble drugs.

## Figures and Tables

**Table 1:** Overview of the investigated drug products, administered dose, and formulation type.

Drug	Trade Name	Dose [mg]	Formulation Type
Aprepitant	Emend ^®^ (MSD, Munich, Germany)	125	Nanocrystal
Celecoxib	Celebrex ^®^ (Pfizer, Vienna, Austria)	200	Microcrystal
Fenofibrate	Lipidil ^®^ (Viatris, Bad Homburg, Germany)	200	Microcrystal
Itraconazole	Sempera 7 ^®^ (JANSSEN-CILAG GmbH, Neuss, Germany)	100	Amorphous solid dispersion
Nimodipine	Nimotop ^®^ (Bayer AG, Leverkusen, Germany)	30	Amorphous solid dispersion
Ritonavir	Norvir ^®^ (AbbVie, Wiesbaden, Germany)	100	Amorphous solid dispersion

**Table 2:** Physicochemical characterization of the poorly soluble drug actives in six investigated drug products: pKa (A*for acid), solubility (S), LogP, fraction absorbed (Fa), and calculated human intestinal effective permeability (P_eff_) values.

Parameter	Aprepitant	Celecoxib	Fenofibrate	Fenofibric acid	Itraconazole	Nimodipine	Ritonavir
pKa	2.8	10.7 (A*)	N/A	4.0 (A*)	3.8	2.6	1.9 2.5
S (0.1N HCl) [µg/mL]	62.0	2.67	0.62	N/A	6.1	3.21	382.8
S (6.8N Buffer) [µg/mL]	1.39	1.76	1.08	1.04 × 10^3^	0.88	2.90	0.96
S (FaSSIF-V2) [µg/mL]	14.0	4.52	1.03	N/A	0.60	5.18	4.3
Log P	4.8	3.7	5.4	3.0	5.4	3.5	4.4
Fa	0.59	0.39	0.46	N/A	0.16	0.035	0.80
P_eff_ [cm/min]	1.67 × 10^−3^	3.71 × 10^−4^	1.64 × 10^−3^	8.78 × 10^−5^	8.13 × 10^−4^	6.00 × 10^−4^	3.30 × 10^−4^

N/A: not available.

## Data Availability

All data presented in this study are available in the research article.
